# Supplementary material for: Immune modulatory effect of a novel 4,5-dihydroxy-3,3´,4´-trimethoxybibenzyl from Dendrobium lindleyi
Source: PLoS One. 2020 Sep 1;15(9):e0238509. doi: 10.1371/journal.pone.0238509 (PMC7462310; doi:10.1371/journal.pone.0238509)
Supplement: Attachment — Submitted filename: Comments to the Authors.pdf [file pone.0238509.s001.pdf]

Comments to the Authors: (PONE-D-20-02679).
Journal: PLOS ONE

Manuscript Number: PONE-D-20-02679

Title: "Immune modulatory effect of a novel 4,5-dihydroxy-3,3´,4´-trimethoxybibenzyl from Dendrobium lindleyi"

Reviewer's comment

The fact is that macrophages, an important population of cells, are involved primarily in defense against infectious disease and play a central role in maintaining all tissues in a healthy state.On the other hand, macrophages also have a multifaceted role in diseases such as cancer, Alzheimer disease, multiple sclerosis and type 1 diabetes… I think that the topic is interesting, the introduction is sufficient to understand the issue, but that the results within the text should be confirmed by statistics. The methods are well described as well as the presentation of the obtained results (Figures). However, I would like to see more data confirming the macrophage function after treatment with isolated components from a Thai orchid *Dendrobium lindleyi* Steud such as phagocytosis ability, confirmation of cytotoxicity in co-culture with tumor cells and measurement of NO and H_2O_2 levels as well as confirmation of anti-inflammatory effect of components through COX-2 inhibition and LOX activities. I believe that confirming the functional ability of macrophages could better link the importance of secretion of individual cytokines with respect to cytokine pleiotropy and redundancy and better and more clearly confirm their immunomodulatory role in health or disease. By confirming the functional capacity of macrophages, the whole research would confirm the greater possibility of application of these components and better justify the mentioned project in plant protection. Such a presentation gives only possible assumptions without confirming the true immunomodulatory role of the isolated components.

Second, minor problems, there are some mistakes about editorial handling, e.g. % must be written close the number. Please harmonize the way of writing degree C. SI units should be used… *(e.g.* µg/ml should be µg/mL). Please separate the SI unit from the number. In vitro and in vivo should be written in italic...... . Statistical value P should be written in italic and with a capital letter. Abbreviations must be explained the first time they are used, both in the Abstract and again in the main text.
